# Selective suppression of {112} anatase facets by fluorination for enhanced TiO_2_ particle size and phase stability at elevated temperatures†

**DOI:** 10.1039/d1na00528f

**Published:** 2021-09-03

**Authors:** Emerson C. Kohlrausch, Roberto dos Reis, Rhys W. Lodge, Isabel Vicente, Alexandre G. Brolo, Jairton Dupont, Jesum Alves Fernandes, Marcos. J. L. Santos

**Affiliations:** Instituto de Química – UFRGS 91501-970 Porto Alegre RS Brazil mjls@ufrgs.br; School of Chemistry, University of Nottingham, University Park Nottingham NG7 2RD UK jesum.alvesfernandes@nottingham.ac.ik; Department of Materials Science and Engineering, Northwestern University Evanston Illinois 60208 USA; Unitat de Tecnologíe Químiques, EURECAT Tarragona 43007 Spain; Department of Chemistry, University of Victoria P. O. Box 3065 V8W 3V6 BC Canada

## Abstract

Generally, anatase is the most desirable TiO_2_ polymorphic phase for photovoltaic and photocatalytic applications due to its higher photoconductivity and lower recombination rates compared to the rutile phase. However, in applications where temperatures above 500 °C are required, growing pure anatase phase nanoparticles is still a challenge, as above this temperature TiO_2_ crystallite sizes are larger than 35 nm which thermodynamically favors the growth of rutile crystallites. In this work, we show strong evidence, for the first time, that achieving a specific fraction (50%) of the {112} facets on the TiO_2_ surface is the key limiting step for anatase-to-rutile phase transition, rather than the crystallite size. By using a fluorinated ionic liquid (IL) we have obtained pure anatase phase crystallites at temperatures up to 800 °C, even after the crystallites have grown beyond their thermodynamic size limit of *ca.* 35 nm. While fluorination by the IL did not affect {001} growth, it stabilized the pure anatase TiO_2_ by suppressing the formation of {112} facets on anatase particles. By suppressing the {112} facets, using specific concentrations of fluorinated ionic liquid in the TiO_2_ synthesis, we controlled the anatase-to-rutile phase transition over a wide range of temperatures. This information shall help synthetic researchers to determine the appropriate material conditions for specific applications.

## Abstract 2

Fluorinated ionic liquids are used to finely control the exposure of {112} facets on the TiO_2_ surface enabling an exquisite temperature control of the anatase-to-rutile phase transition.

## Graphical abstract

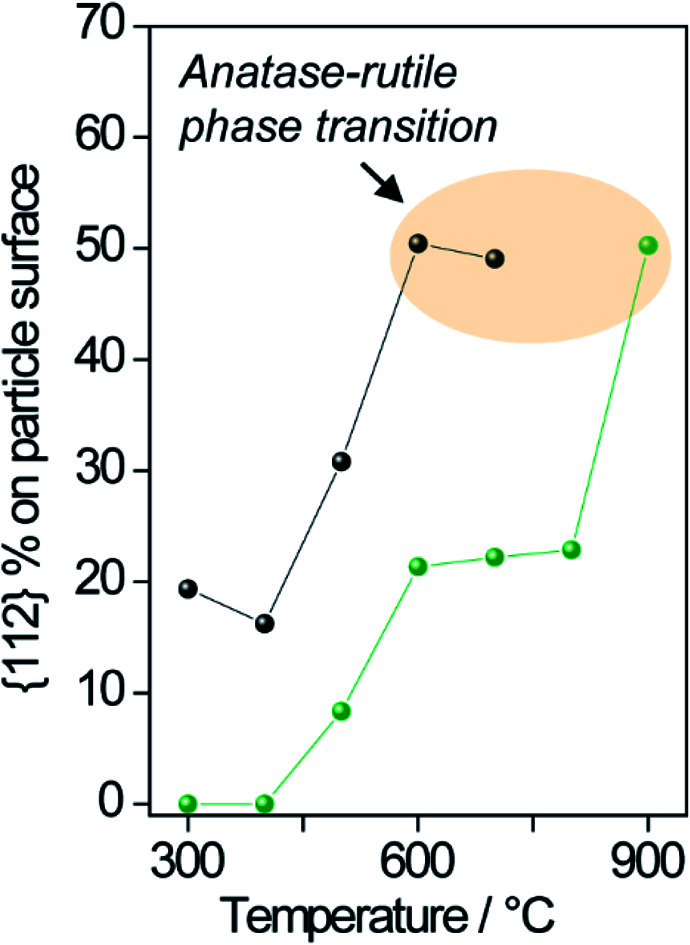

## Introduction

Surface chemistry plays a crucial role in the properties of solid-state materials, especially in nanoparticles where high surface-area-to-volume ratios amplify the unique physicochemical characteristics of highly energetic surface atoms.^1^ Significant efforts have been devoted to controlling nanoparticle facets to produce materials with predictable optical, electrical, and catalytic properties.^2–4^ The surface energy of these facets contributes to the minimization of the total surface energy of the particle during crystal growth, which can be controlled, to a certain degree, by certain synthetic approaches.^5–7^

One of the most explored approaches to control the size, shape, and catalytic properties of TiO_2_ is through the incorporation of dopants, such as transition metals (Fe, Ni, Co) or non-metals (N, F, S).^8–16^ The effect of fluorine on the size, phase, and morphology of TiO_2_ particles has been widely explored and hydrofluoric acid has been found to be the key reactant to obtain nanoparticles with their surface dominated by exposed {001} facets.^17^ Urea and EDTA have also been found to drive the formation of TiO_2_ nanoparticles with large {001} facets.^18–20^ Adjusting the fluoride/titanium molar ratio controlled the size of TiO_2_ sheets with predominant {001} facets which was shown to improve the photodegradation efficiency of organic dyes.^21^ {001} facets on uniform anatase TiO_2_ single crystals were also selectively grown using hydrofluoric acid as a morphology controlling agent.^22^ More recently, another study of TiO_2_ growth using HF showed that whilst fluorine was responsible for the anatase morphology with exposed low-index facets, H^+^ was responsible for increasing the percentage of {001} facets.^23^ When TiO_2_ was synthesized in fluorinated ionic liquids, it preferentially formed the brookite phase, rather than anatase, above a certain concentration of [BF_4_]^−^ anions. Subsequent thermal treatment led to a transition from brookite to anatase that was accompanied by the release of fluorine, through which the authors concluded that fluorine was responsible for stabilizing the brookite phase.^24^

Of the notable facets of anatase, the {112} facet is important because it plays a crucial role in the anatase-to-rutile phase transitions. Penn *et al.* have demonstrated that rutile nucleate at {112} twin anatase interfaces.^25^ Recently, Zhu *et al.*^26^ have studied the pathways of surface restructuring, showing that {112} undergoes reconstruction that can lead to a new phase propagating into the anatase bulk, while the reconstruction of (001), (100), (101), and other planes only takes place at the surface. Therefore, they concluded that only the {112} in anatase is responsible for the initial nucleation that will allow anatase-to-rutile phase transition.

Furthermore, the TiO_2_ crystallite size is dependent on the thermodynamic stability of each polymorphic phase. For crystallites smaller than 35 nm, the anatase phase is more stable than rutile, and an anatase-to-rutile phase transition only starts after the thermodynamic critical size is reached (>35 nm) which usually occurs at temperatures above 500 °C.^27–30^ However, the growth of pure anatase phase TiO_2_ nanoparticles larger than 35 nm at temperatures above 500 °C has yet to be reported as they are thermodynamically unfavorable.^31–33^

In this work, we attempted to synthesize pure anatase phase TiO_2_ nanoparticles larger than 35 nm in a fluorinated ionic liquid, 1-butyl-3-methylimidazolium tetrafluoroborate (BMIm·BF_4_), at temperatures up to 800 °C and compare their efficiency with that of pure TiO_2_ nanoparticles when applied to dye-sensitized solar cells (DSSCs). By using XRD diffraction patterns from thermally treated nanoparticles in a wide range of temperature, we have used the Wulff construction to extract meaningful information about facet growth on the TiO_2_ particles.^34–36^

## Experimental section

### Synthesis of TiO_2_

TiO_2_ nanoparticles were synthesized by adding acetic acid (5.7 mL) to titanium isopropoxide (15 mL) under constant stirring at 25 °C.^37^ The solution was stirred for 15 min and poured into deionized water (70 mL). The mixture was stirred for one hour at room temperature to complete the hydrolysis. The ionic liquid (IL) BMIm·BF_4_ (1% or 10% w/w, with respect to deionized water) was then added to the solution, in addition to nitric acid (63%, 1 mL), before being stirred for 8 hours at 80 °C. Finally, the mixture was transferred to an autoclave and heated at 230 °C for 12 hours. All samples were subsequently rinsed with water (3 × 50 mL) followed by ethanol (3 × 50 mL). The samples were labelled as TiO_2_ (no addition of IL), TiO_2_/IL 1% (1% (w/w) of the ionic liquid) and TiO_2_/IL 10% (10% (w/w) of the ionic liquid). The samples were thermally treated at 300, 400, 500, 600, 700, 800 and 900 °C for 3 hours in air.

### Characterization

The morphology, size and structural characteristics of the as-synthesized TiO_2_ nanoparticles were observed by transmission electron microscopy performed with a Libra Zeiss 120 and a Philips CM300. SEM images were obtained using a JEOL 7100F Field-Emission Gun Scanning Electron Microscope (FEG-SEM). A working distance of 10 mm was maintained with acquisitions utilizing a beam voltage of 15 kV. For analysis, a small amount of the sample was deposited onto double-sided carbon tape mounted on a stub, followed by sputter-coating with iridium (5 nm thickness) to make the sample conductive. X-ray powder diffraction (XRD) patterns were obtained using a Siemens D5000 diffractometer with Cu-Kα radiation (*λ* = 1.5418 Å) in a 2*θ* range from 10 to 90° with a step size of 0.05° and time of 1 s per step.

### Wulff grain construction

The Wulff grain construction was obtained by inputting the Miller indices and respective crystallite size for the {*hkl*} plane families {101}, {103}, {004} and {112} from the anatase phase with the auxiliary of VESTA software. The size of each family's planes was obtained using the Scherrer equation from XRD patterns obtained in the present work. Wulff construction was performed using the atomic position set and the space group of the anatase structure *I*4_1_/*amd*, no. 141. The unit cell is defined by the lattice vectors *a* and *c* and contains two TiO_2_ units with Ti ions at 4b Wyckoff positions (0, 1/4, 3/8) and (0, 3/4, 5/8) and O ions at 8e Wyckoff positions (0, 1/4, *u*), (0, 3/4, 1/4 + *u*), (1/2, 1/4, −*u* + 1/2) and (1/2, 3/4, 1/4 − *u*).^38,39^

### DSSC assembly and measurements

The procedure used to assemble the DSSCs followed a previous literature method.^37^ The characterization and performance of the DSSCs were evaluated by current *versus* potential measurements, and carried out using a 300 W xenon arc lamp and an AM1.5 filter. The power of the simulated light was calibrated to 100 mW cm^−2^ and recorded by a picoamperimeter (Keithley, model 2400).

## Results and discussion

**Fig. 1:**
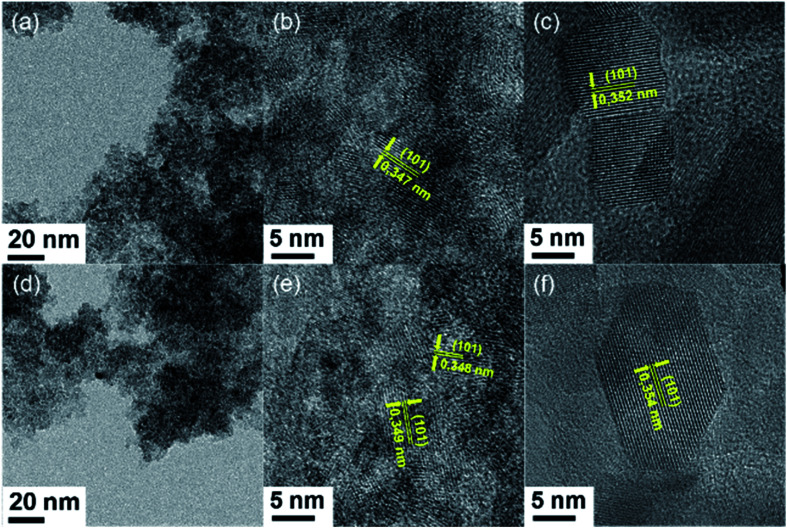
TEM images obtained after hydrolysis at 80 °C from TiO_2_/IL 1% (a and b) and TiO_2_/IL 10% (d and e). HRTEM images obtained after hydrothermal reaction at 230 °C from TiO_2_/IL 1% (c) and TiO_2_/IL 10% (f).

HR-TEM investigations of TiO_2_, TiO_2_/IL 1% and TiO_2_/IL 10% were carried out after different synthetic steps. TEM images were acquired for all samples after the hydrolysis step and, while no formation of anatase seeds was observed from TiO_2_, Fig. 1 shows the formation of anatase nanoseeds from TiO_2_/IL 1% and TiO_2_/IL 10% (Fig. 1a, b and d, e, respectively).^40–43^ After the autoclave step at 230 °C, crystalline particles were obtained for all the samples, and no significant variation in morphology, crystalline structure, or size distribution was observed (Fig. S1 and S2†).

**Fig. 2:**
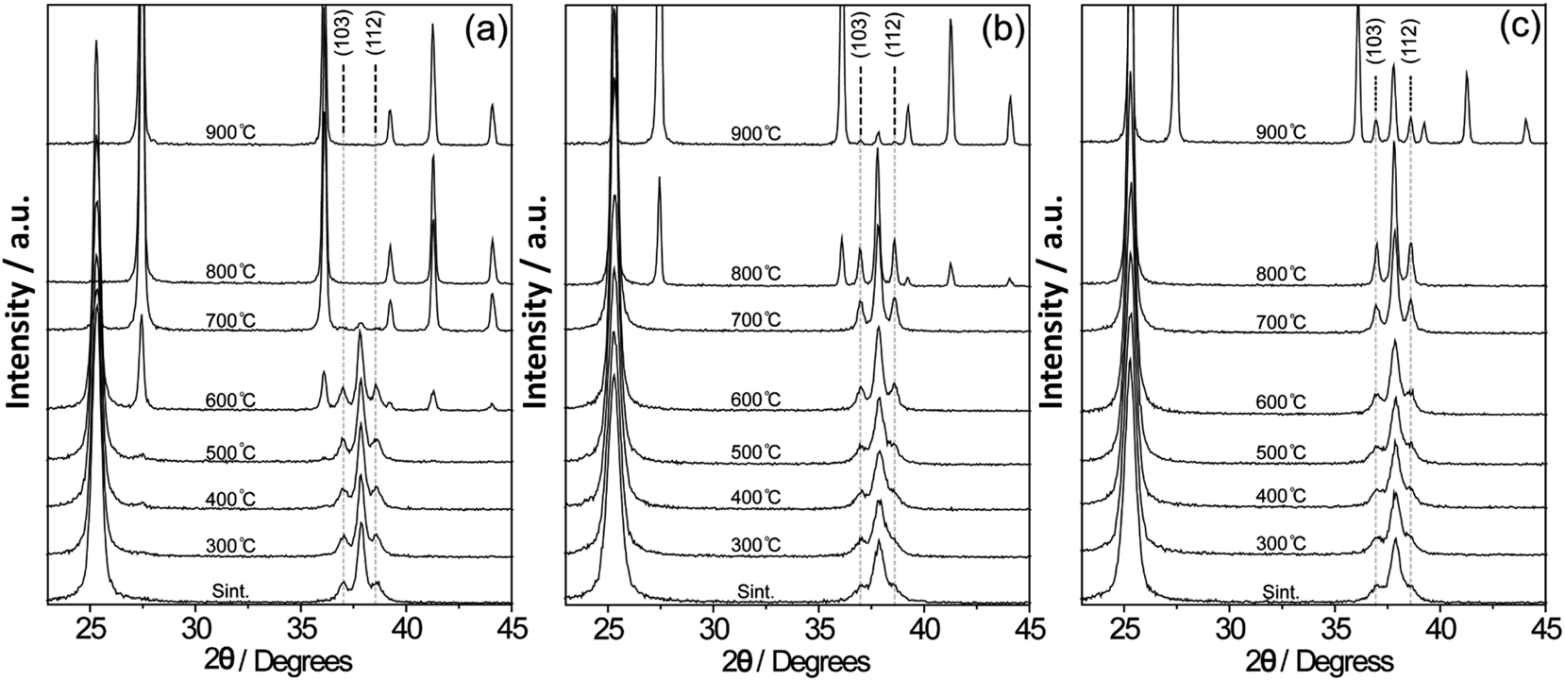
(a–c) X-ray diffraction patterns of the as-synthesized and thermally treated samples from 300 °C to 900 °C for TiO_2_, TiO_2_/IL 1%, and TiO_2_/IL 10%, respectively. Temperature dependence of the peaks related to the (103) and (112) planes.

**Fig. 3:**
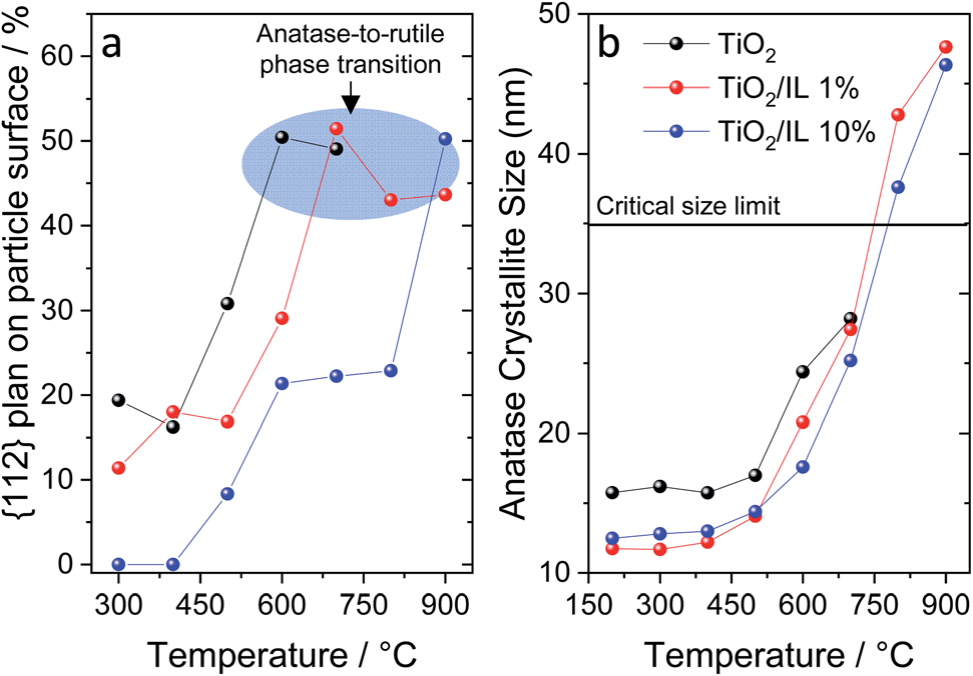
(a) Percentage of {112} planes on the particle surface as a function of temperature highlighting the suppression of this facet by the IL compared to pure TiO_2_. The data were extracted from the Wulff construction (see details in ESI Table S7†). (b) Anatase crystallite size for all samples, from the {101} planes (Table S3†), as a function of the temperature highlighting the crystallite critical size limit.

To track TiO_2_ phase transitions, and to evaluate the thermal stability of the anatase crystals, the samples were thermally treated at 300, 400, 500, 600, 700, 800 and 900 °C (Fig. 2 and S3†). For TiO_2_, the phase transition of anatase-to-rutile began at 400 °C with a small diffraction peak at *ca.* 27.4°. As the temperature rose to 600 °C, a sharper, defined diffraction peak at *ca.* 27.4° was observed (Fig. 2a). The calculated TiO_2_ crystallite sizes at 600 °C were 24.4 and 36.2 nm for anatase and rutile, respectively (Fig. 3b and Table S1†). A complete conversion to rutile took place between 700 and 800 °C resulting in rutile crystallites 43.0 nm in size. At 700 °C, only a small number of anatase particles with a crystallite size of 28.2 nm were observed. For TiO_2_/IL 1% (Fig. 2b), the anatase-to-rutile phase transition started at *ca.* 800 °C with anatase presenting a crystallite size of 42.8 nm and an incomplete conversion of anatase-to-rutile being observed even at 900 °C with anatase crystallites of 47.6 nm still present (Table S1†). In the TiO_2_/IL 10% sample (Fig. 2c), the anatase-to-rutile phase transition only started at 900 °C and again showed anatase crystallites (47.5 nm) beyond their thermodynamic limit of 35 nm. Raman spectroscopy measurements revealed that the temperature required to promote the solid-to-solid phase transition from anatase-to-rutile was dependent on the concentration of fluorine (Fig. S4†). For TiO_2_ thermally treated at temperatures higher than 600 °C, the rutile phase was predominant over anatase; however, for TiO_2_/IL 1% this predominance was only observed at 900 °C and for TiO_2_/IL 10% anatase was the main phase even at 900 °C. UV-Vis measurements showed a significant redshift for the fluorinated samples, when compared with TiO_2_, at the temperature at which the phase transition took place (Fig. S5†).^44–47^

As discussed earlier, the anatase-to-rutile phase transition temperature can be affected or controlled by how efficiently {112} twin boundaries interact with each other.^25,26^ Therefore, we evaluated the effect of fluorine on the {112} facet growth by comparing the equivalent diffraction peaks related to the {112} plane at 38.60° and to the {103} plane at 36.95° (PDF number # 21-1272) (see Tables S2 and S3†).^48^ For the fluorine–TiO_2_ samples, the relative areas of the diffraction peaks ({112} and {103}) became nearly equivalent only at temperatures at which the material was about to undergo the anatase-to-rutile phase transition, while for pristine TiO_2_ they remained similar to those for the synthesis at 600 °C. These results demonstrated that fluorine plays a critical role in suppressing the formation of {112} planes, thus delaying the anatase-to-rutile transition. The crystallite sizes for the {101}, {103}, {004} and {112} planes for TiO_2_, TiO_2_/IL 1% and TiO_2_/IL 10% are presented in Tables S4–S6.† Although many reports in the literature have shown the use of fluoride to drive the preferential growth of {001} facets in TiO_2_,^22,49,50^ in the present work we have no clear evidence about the influence of the fluorinated ionic liquid on the {001} planes. In fact, neither hydrofluoric acid nor titanium tetrachloride was used in the synthesis and the F/Ti molar ratio in both samples was smaller than that commonly used to drive the preferential growth of {001} facets. We suggest that the amount of fluorine used to obtain TiO_2_/IL 1% and TiO_2_/IL 10% is enough to suppress the {112} planes, but not to affect the {001} growth.

Wulff grain construction was performed to confirm the effect of fluorine on the {112} plane at different temperatures (Fig. 3a and S6, and Tables S7–S10†).^51^ Wulff principles are widely used in grain growth to determine which crystal geometrical shape has the minimum surface energy. The morphological equilibrium of the crystal corresponds to the minimization of the surface energy; from a thermodynamic perspective, the equilibrium crystal shape corresponds to the lowest free energy of the particle under specific conditions.^52,53^Fig. 3a shows the Wulff construction and highlights the {112} content, as a percentage, at which the anatase-to-rutile phase transition started. For pristine TiO_2_, the contribution of the {112} facets increased from *ca.* 15% to 50% from 400 to 600 °C. TiO_2_/IL 1% presented a similar trend, albeit from 500 to 700 °C instead. For TiO_2_/IL 10%, three prominent features were observed: (i) the complete suppression of the {112} facets up to 400 °C; (ii) a contribution of only 20–25% of the {112} facets up to 800 °C; and (iii) a *ca.* 50% contribution at 900 °C, the temperature at which the anatase-to-rutile phase transition started for TiO_2_/IL 10%. Additionally, Fig. 3b shows the crystallite sizes obtained from XRD measurements at different temperatures, highlighting the size limit within which anatase is thermodynamically stable. While a maximum size of *ca.* 28.2 nm was observed for TiO_2_, the samples modified with fluorinated ionic liquid, TiO_2_/IL 1% and TiO_2_/IL 10%, exhibited crystallites of *ca.* 48 nm that surpass the thermodynamic limit. These results strongly suggested that achieving a specific fraction of the {112} facets might be the threshold limiting step for anatase-to-rutile phase transition and not the crystallite size itself.

**Fig. 4:**
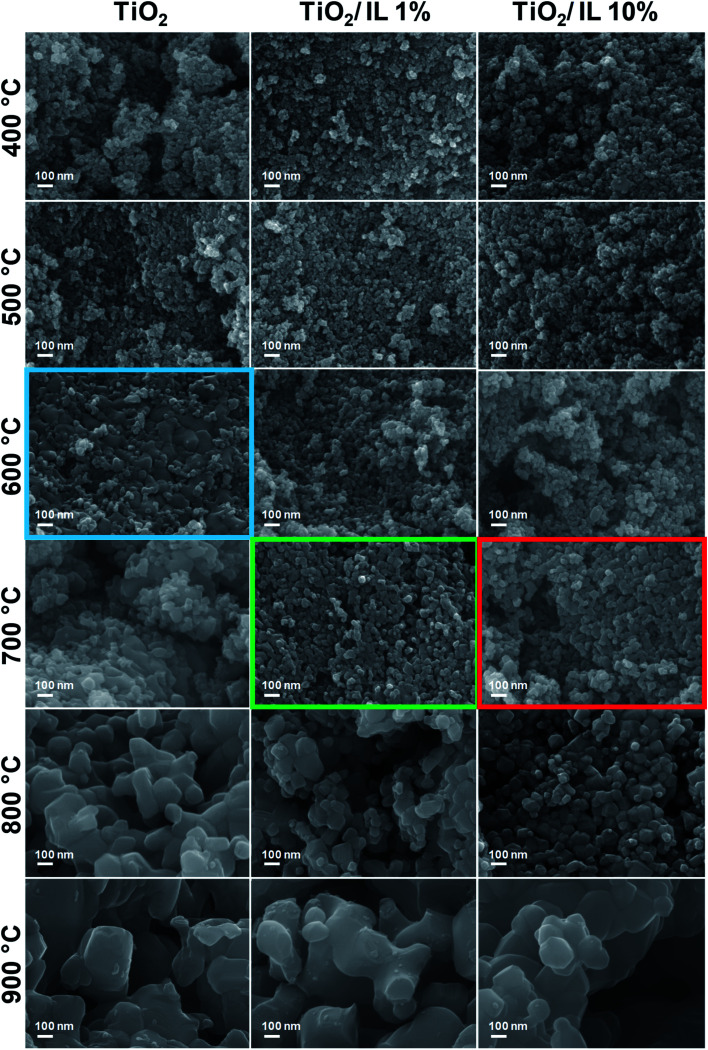
FEG-SEM images of TiO_2_, TiO_2_/IL 1%, and TiO_2_/IL 10% thermally treated from 300 °C to 900 °C. These images clearly show that TiO_2_ morphological changes occur at different temperatures for the fluorinated TiO_2_ nanoparticles (green and red boxes) when compared with pure TiO_2_ (blue box).

FEG-SEM of TiO_2_, TiO_2_/IL 1% and TiO_2_/IL 10% was performed to investigate the morphological changes before and after thermal treatment (Fig. 4). For pristine TiO_2_, a sharp change in nanoparticle morphology was observed at 600 °C (blue box, Fig. 4), at which the sintering of relatively small TiO_2_ nanoparticles led to the formation of much larger nanoparticles; this agreed with the anatase-to-rutile phase transition observed in XRD and Raman spectroscopy measurements. However, for TiO_2_/IL 1% and TiO_2_/IL 10% the sintering and formation of larger anatase nanoparticles were observed at 700 °C (green and red boxes, respectively, Fig. 4), which was a lower temperature than that observed for the anatase-to-rutile transition by XRD and Raman spectroscopy. In contrast to some previous reports, this demonstrated that the anatase-to-rutile phase transition limiting step is directly related to {112} facet concentration rather than a critical crystallite size of 35 nm.

EDX spectroscopy and XPS (Fig. S7–S10 and Table S3†) revealed the presence of fluorine in the samples prepared with ionic liquid prior to and after thermal treatment, while neither boron nor nitrogen were observed. HR-XPS of the F 1s region from the as-synthesized TiO_2_/IL 1% and TiO_2_/IL 10% showed a peak at 684.6 eV which was associated with adsorbed F^−^ ions on the TiO_2_ surface (Fig. S10b and S10c†).^54^ However, after thermal treatment at 500 °C, an additional peak at 687.8 eV was observed which was attributed to the incorporation of fluorine atoms into the TiO_2_ structure (Figure S10e and S10f†). This suggested that the fluorine ions were coordinating to surface Ti^4+^ and Ti^3+^ ions, impacting the anatase-to-rutile phase transition.^55,56^

Photocurrent *versus* voltage (*I*–*V*) measurements of DSSCs assembled with TiO_2_, TiO_2_/IL 1%, and TiO_2_/IL 10% were carried out (Fig. S11†) and the following parameters were measured from the assembled devices: the short-circuit current (*I*_sc_), open-circuit voltage (*V*_oc_), Fill Factor (FF) and efficiency (*η*) (Table 1). The power conversion efficiencies (under AM 1.5G illumination) of standard TiO_2_, TiO_2_/IL 1%, and TiO_2_/IL 10% were 5.50%, 5.96%, and 6.35%, respectively. The 15% enhancement in efficiency found for TiO_2_/IL 10%, when compared to TiO_2_, can be ascribed to the red-shift shown by the fluorinated samples. Additionally, it could be due to the suppression of rutile phase growth for the TiO_2_ fluorinated samples during the sintering of the device assembly (Fig. S12†).^57^ This may lead to a decrease in the density of defects in the fluorinated samples when compared to pristine TiO_2_, which can act as recombination centers for photogenerated electron–hole pairs.^58–60^

**Table 1:** Electrical parameters, FF and efficiency obtained from the assembled DSSCs

Sample	*I*_sc_ (mA)	*V*_oc_ (V)	*I*_max_ *V*_max_	FF	*η*
TiO_2_	14.50	0.71	5.50	55%	5.50%
TiO_2_/IL 1%	14.75	0.71	5.96	57%	5.96%
TiO_2_/IL 10%	15.12	0.73	6.35	58%	6.35%

## Conclusions

An ionic liquid (BMIm·BF_4_) was used in the hydrothermal synthesis of TiO_2_, resulting in fluorinated anatase phase nanoparticles greater in size than the previously determined 35 nm size limit. Fluorine favored the formation of the anatase phase at very low temperatures and contributed to the maintenance of this phase by hindering the growth of the {112} planes. This delayed the anatase-to-rutile phase transition, requiring much higher temperatures than usually observed for TiO_2_. Wulff construction along with analyses by XPS, XRD, and Raman spectroscopy showed that the {112} facet played the main role in the anatase-to-rutile phase transition of TiO_2_. These results strongly suggested that achieving a specific fraction of the {112} facets might be the threshold limiting step for anatase-to-rutile phase transition. Our findings demonstrate that the formation of {112} facets on the TiO_2_ surface can be finely tuned by altering the concentration of fluorinated ionic liquid in the TiO_2_ synthesis, enabling precise control of the anatase-to-rutile phase transition for a wide range of temperatures. DSSCs assembled with mesoporous layers based on TiO_2_/IL 1% and TiO_2_/IL 10% presented an improvement of the photocurrent and fill factor, and 15% greater efficiency when compared to pristine TiO_2_.

## Conflicts of interest

There are no conflicts to declare.

## Supplementary Material

NA-003-D1NA00528F-s001
