# Supplementary material for: Selective suppression of {112} anatase facets by fluorination for enhanced TiO2 particle size and phase stability at elevated temperatures
Source: Nanoscale Adv. 2021 Sep 3;3(21):6223–30. doi: 10.1039/d1na00528f (PMC9419165; doi:10.1039/d1na00528f)
Supplement: NA-003-D1NA00528F-s001 [file NA-003-D1NA00528F-s001.pdf]

Electronic Supplementary Material (ESI) for Nanoscale Advances.
This journal is © The Royal Society of Chemistry 2021

Selective Suppression of {112} Anatase Facet by Fluorination for Enhanced TiO_2 Particle Size and Phase Stability at Elevated Temperatures

Emerson C. Kohlrausch,[a,b] Roberto dos Reis,[c] Rhys W. Lodge,[b] Isabel Vicente,[d] Alexandre G. Brolo,[e] Jairton Dupont,[a] Jesum Alves Fernandes.[b]* and Marcos. J. L. Santos[a]*

[a] *Instituto de Química – UFRGS, 91501-970, Porto Alegre, RS, Brazil*
[b] *School of Chemistry, University of Nottingham, University Park, Nottingham, NG7 2RD, United Kingdom*
[c] *Department of Materials Science and Engineering, Northwestern University, Evanston, Illinois 60208, United States*

[d] *Unitat de Tecnologíe Químiques, EURECAT, Tarragona, 43007 Spain*

[e] *Department of Chemistry, University of Victoria, P.O. Box 3065, V8W 3V6, BC, Canada*

Experimental section

Characterization

The morphology size and structural characteristics of as-synthesized TiO_2 nanoparticles was observed by Transmission electron microscopy performed with a Libra Zeiss 120 and a Philips CM300. SEM images were obtained using a JEOL 7100F Field-Emission Gun Scanning Electron Microscope (FEG-SEM). A working distance of 10 mm was maintained with acquisitions utilizing a beam voltage of 15 kV. To analyze, a small amount of the sample was deposited onto a double-sided carbon tape mounted on a stub, followed by sputter-coating with iridium (5 nm thickness) to make the sample conductive.

Absorption spectra from the thermally treated samples were obtained at diffuse reflection mode using a Shimadzu UV-2450PC spectrophotometer with integrating sphere ISR-2200, at room temperature.

X-ray powder diffraction (XRD) patterns were obtained using a Siemens D5000 diffractometer with Cu-Kα (λ= 1.5418 Å) in a 2θ range from 10 to 90° with a step size of 0.05° and time of 1 s per step.

Raman spectroscopy was performed using an InVia Renishaw microscope (Renishaw Inc., Hoffman Estates, IL) with a 5× dry objective (Leica Microsystems, Wetzlar, Germany) and 1200 lines/mm diffraction grating. A 633 nm Helium Neon Laser (Renishaw plc, Transducer

Systems Division, Gloucestershire, UK)) was used for sample excitation. Spectra were acquired with a 5 s exposure and a laser power of ~1 mW at the sample. Laser power through the objective was measured using a Coherent FieldMax-TOP Laser Power/Energy Meter (Coherent Inc., Portland, Oregon, USA).

X-ray photoelectron spectroscopy (XPS) measurements were performed using a Kratos AXIS Ultra DLD instrument. The chamber pressure during the measurements was $5 \times 10-9$ Torr. Wide energy range survey scans were collected at pass energy of 80 eV in hybrid slot lens mode and a step size of 0.5 eV. High-resolution data on the C 1s, O 1s, N 1s, B 1s, F 1s, and Ti 2p photoelectron peaks were collected at a pass energy 20 eV over energy ranges suitable for each peak, and collection times of 5 min, step sizes of 0.1 eV. The charge neutralizer filament was used to prevent the sample from charging over the irradiated area. The X-ray source was a monochromated Al Kα emission, run at 10 mA and 12 kV (120 W). The energy range for each 'pass energy' (resolution) was calibrated using the Kratos Cu $2p_{3/2}$, Ag $3d_{5/2}$, and Au $4f_{7/2}$ three-point calibration method. The transmission function was calibrated using a clean gold sample method for all lens modes and the Kratos transmission generator software within Vision II. The data were processed with CASAXPS (Version 2.3.17).

Wulff grain construction

Wulff grain construction was obtained by inputting the miller indices and respective crystallite size for the {hkl} plan families {101}, {103}, {004} and {112} from anatase phase with the auxiliary of VESTA software. The size of each family's plane was obtained by Scherrer equation from XRD patterns obtained in the present work. Wulff construction was performed using the atomic position set and the space group of the anatase structure $I4_1/amd$, N° 141. The unit cell is defined by the lattice vectors a and c and contains two TiO_2 units with Ti ions at *4b Wyckoff* positions (0, 1/4, 3/8), (0, 3/4, 5/8) and O ions at *8e Wyckoff* positions (0, 1/4, u), (0, 3/4, 1/4+u), (1/2, 1/4, -u+1/2) and (1/2, 3/4, 1/4-u). [1,2]

DSSC assembly and measurements

The procedure used to assemble the DSSCs followed a previous literature method [3]. The characterization and the performance of the DSSCs were evaluated by current versus potential measurements, carried out using a 300 W Xenon arc lamp and an AM1.5 filter. The power of the simulated light was calibrated to 100 mW/cm^2 and recorded by a picoamperimeter Keithley, model 2400.

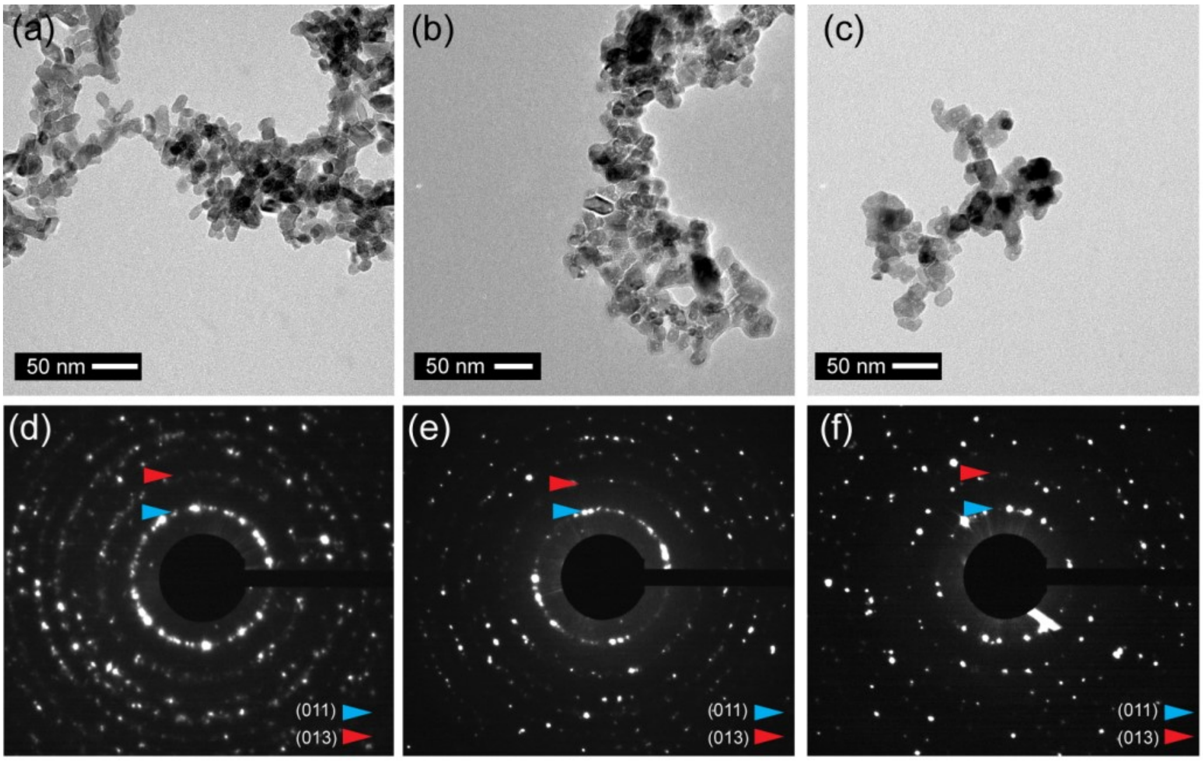

Figure S1: TEM images and electron diffraction patterns of TiO_2 (a and d), TiO_2/IL 1% (b and e) and TiO_2/IL 10% (c and f) after treatment in the autoclave at 230 ºC.

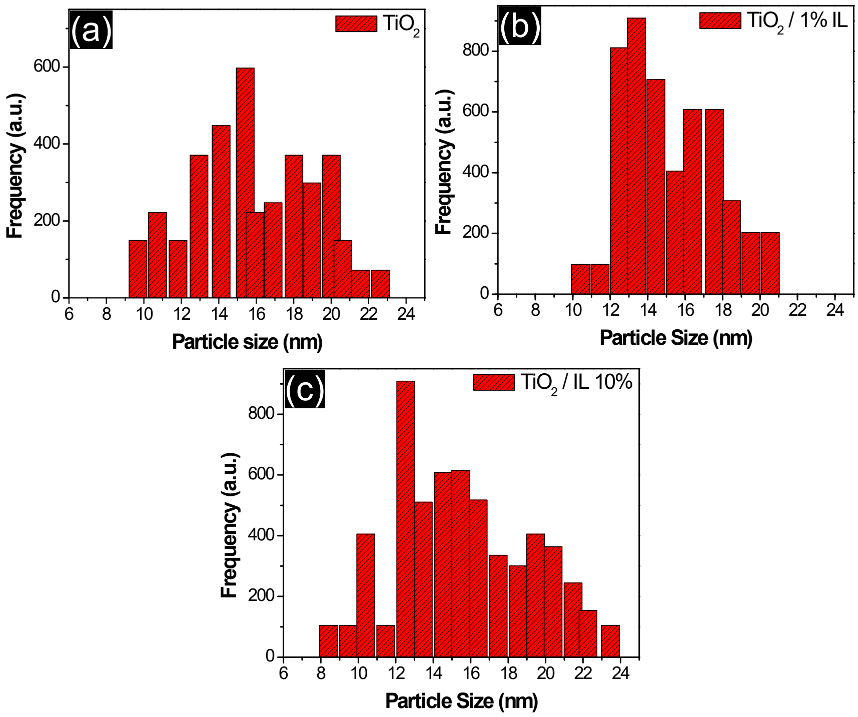

Figure S2: Histograms presenting the mean particle size for: as-synthesized TiO_2 (a), TiO_2/IL 1% (b) and TiO_2/IL 10% (c).

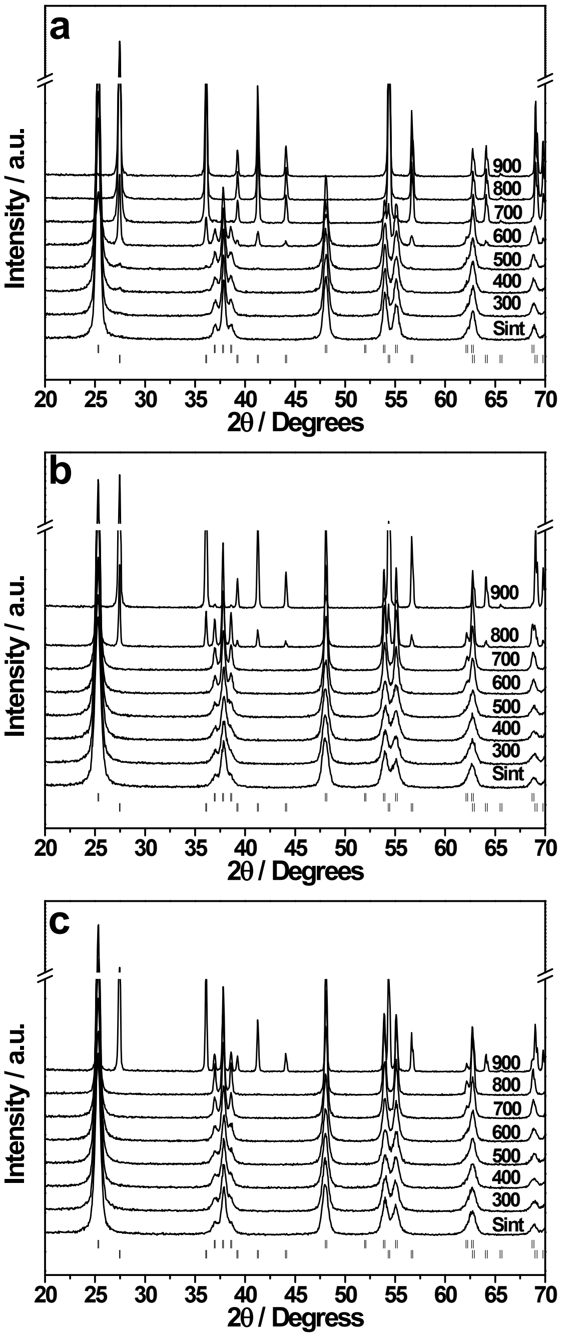

Figure S3: Full range of X-ray diffraction pattern of as-synthesized and thermally treated samples from 300 °C to 900 °C for (a) TiO_2, (b) TiO_2/IL 1%, and (c) TiO_2/IL 10%.

Table S1: Crystallite diameter (nm), from the {101} planes, as a function of thermal treatment temperature obtained from XRD.

Temperature	TiO_2	TiO_2/IL 1%	TiO_2/IL 10%
As sint.	15.7	11.7	12.8
400 °C	15.7	14.0	13.0
500 °C	17.0	14.1	14.4
600 °C	24.4/36.2*	20.8	17.6
700 °C	28.2/43.0*	27.4	25.2
800 °C	--- /45.9*	42.8/49.5*	37.6
900 °C	--- /46.0*	47.6/49.9*	47.5/51.5*

*Crystallite size by rutile phase.

In agreement with XRD measurements, Raman spectroscopy exhibited the typical anatase active modes, B_{1g} (397 cm^{-1}), A_{1g} (514 cm^{-1}), E_g (143 cm^{-1}) and E_g (637 cm^{-1}), revealing that the temperature required to promote solid-to-solid phase transition from anatase-to-rutile is dependent on the presence and the concentration of the ionic liquid. The oxygen vacancies in the TiO_2 matrix was observed by the O-Ti-O bending vibration at higher frequencies (146 cm^{-1}) for the anatase phase, while the E_g mode of rutile (447 cm^{-1}) shifted to lower frequencies [4]. The higher intensity of the vibration modes of anatase and rutile in TiO_2/IL 1% and TiO_2/IL 10%, when compared to pristine TiO_2, can be associated with an increase in crystallinity and crystallite size due to the presence of fluorine [5].

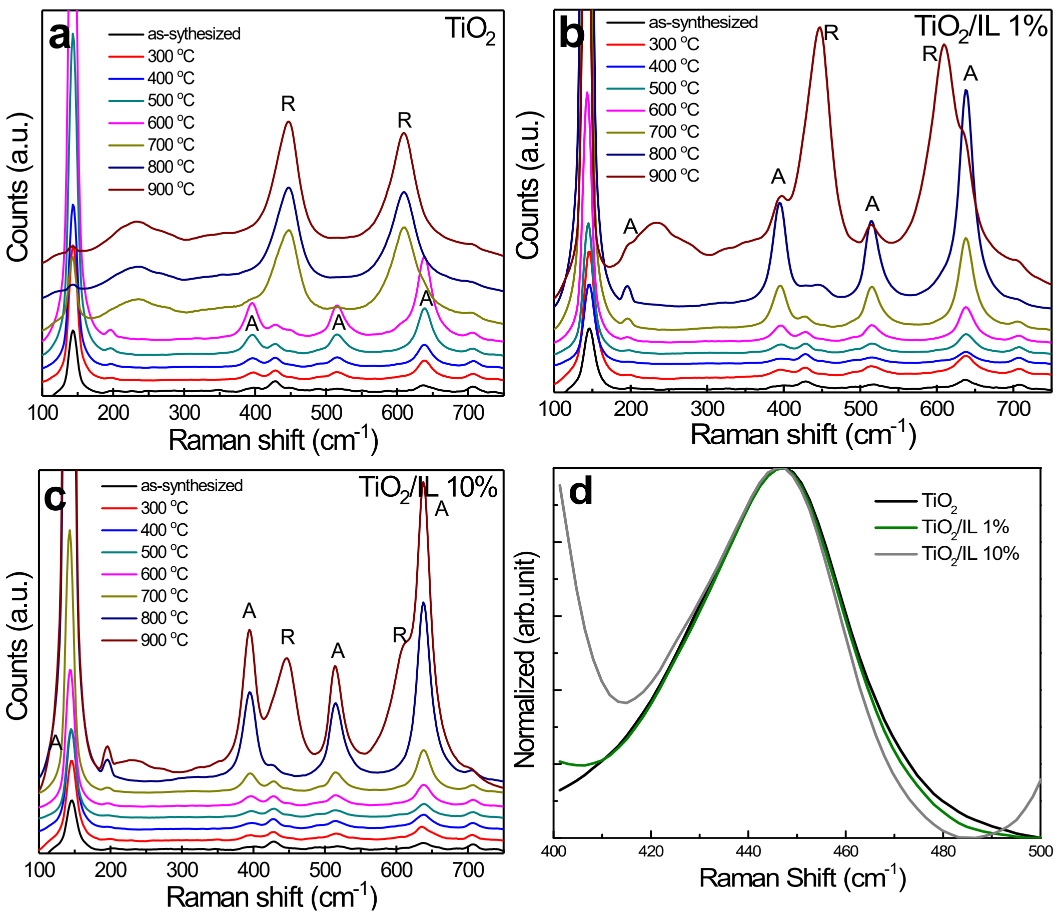

Figure S4: Raman spectra of (a) TiO_2, (b) TiO_2/IL 1%, (c) TiO_2/IL 10% obtained after thermal treatment at 300, 400, 500, 600, 700, 800 and 900 °C and (d) Normalized Raman spectra of TiO_2, TiO_2/IL 1% and TiO_2/IL 10% thermally treated at 900 °C.

The role of fluorine inhibiting the formation of the rutile phase was also confirmed by UV-Vis spectroscopy obtained from the samples thermally treated in different temperatures, showing a similar trend observed in XRD (Figure 2) and Raman (Figure S4). Interestingly, the absorption spectra of as-synthesized TiO_2/IL 1% and TiO_2/IL 10% did not display characteristics of doping, as usually observed in non-metal doped TiO_2 for instance, hence the fluorination has no significant effect on the bandgap of anatase TiO_2 [6-7]. In both vibration modes associated with anatase and rutile phase, no significant shift was observed, which suggested an extremely low concentration of oxygen vacancies in the TiO_2 NPs, corroborating with XPS (Figure S8-S10).

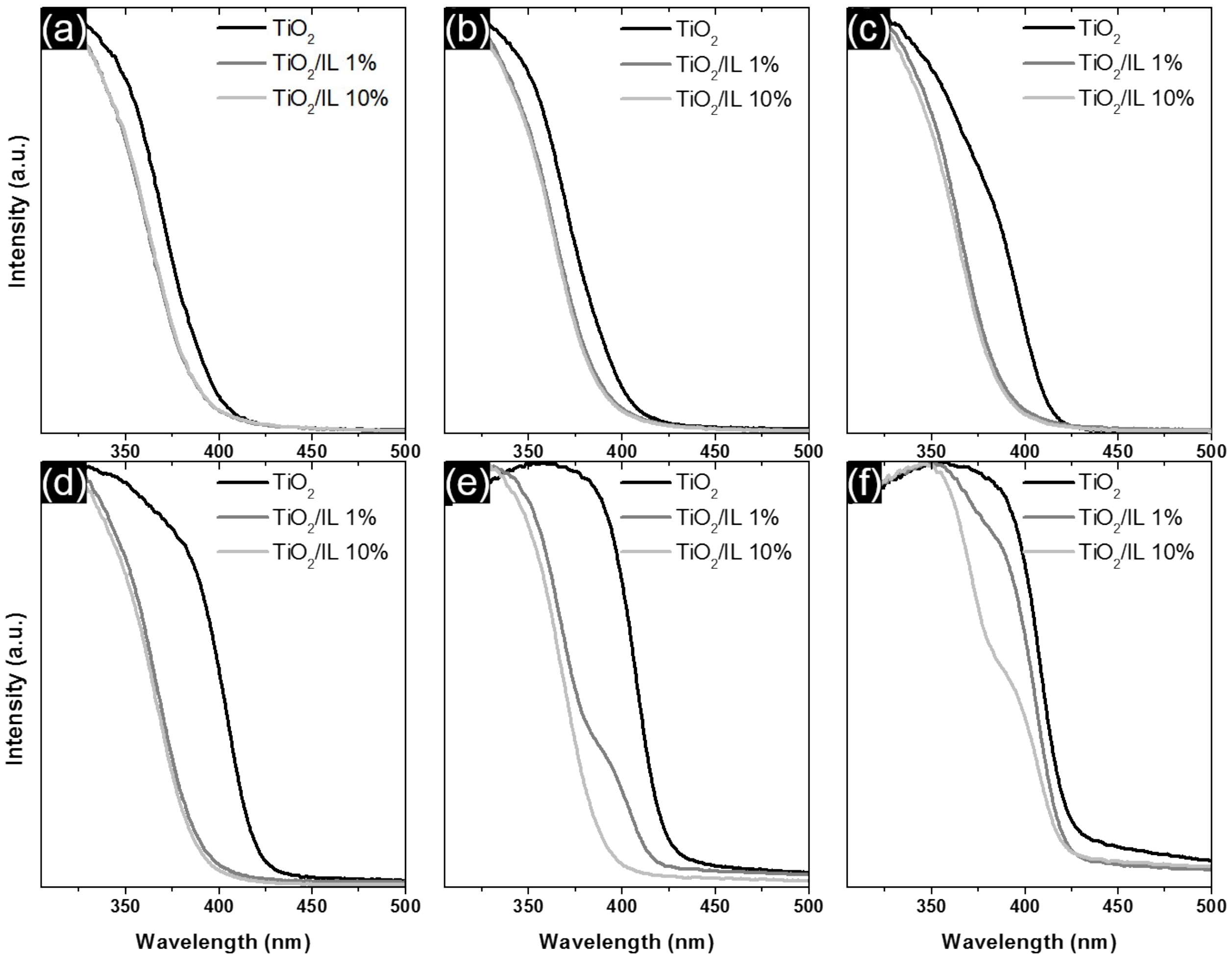

Figure S5: UV-Visible spectra of the samples after thermal treatment at 400 °C (a), 500 °C (b), 600 °C (c), 700 °C (d), 800 °C (e) and 900 °C (f).

Table S2: Area of the diffraction peaks of {103} and {112} for all samples as a function of thermal treatment temperature obtained from XRD.

	TiO_2		TiO_2/IL 1%		TiO_2/IL 10%	
Temperature	{103}	{112}	{103}	{112}	{103}	{112}
As sint.	103.6	105.8	163.5	139.2	128.9	82.5
300 °C	109.4	108.2	127.4	121.8	158.8	134.1
400 °C	104.0	111.3	151.9	137.6	87.9	83.2
500 °C	99.4	119.6	102.8	89.5	107.1	96.5
600 °C	83.7	108.8	116.7	117.9	120.5	118.5
700 °C	64.5	83.6	107.7	123.7	111.6	129.1
800 °C	n.a.	n.a.	97.3	118.9	121.8	122.0
900 °C	n.a.	n.a.	14.4	13.2	43.5	43.0

Table S3: {112}/{103} ratio as a function of thermal treatment temperature obtained from XRD for all samples.

Temperature	TiO_2	TiO_2/IL 1%	TiO_2/IL 10%
As sint.	1.02124	0.85138	0.64003
300 °C	0.98903	0.95604	0.84446
400 °C	1.07019	0.90586	0.94653
500 °C	1.20322	0.87062	0.90103
600 °C	1.29988	1.01028	0.9834
700 °C	1.29612	1.14856	1.15681
800 °C	n.a.	1.22199	1.00164
900 °C	n.a.	0.91667	0.98851

Table S4: Crystallite size (nm) for the {101}, {103}, {004} and {112} planes for the sample TiO_2 as a function of thermal treatment temperature obtained from XRD.

Temperature	{101}	{103}	{004}	{112}
As sint.	15.7	20.5	22.7	17.7
300 °C	15.7	19.5	23.0	21.5
400 °C	16.0	20.9	23.2	19.3
500 °C	17.0	25.2	23.1	19.4
600 °C	24.4	25.2	27.9	25.1
700 °C	28.2	30.9	29.1	28.2
800 °C	n.a.	n.a.	n.a.	n.a.
900 °C	n.a.	n.a.	n.a.	n.a.

Table S5: Crystallite size (nm) for the {101}, {103}, {004} and {112} planes for the sample TiO_2/IL 1% as a function of thermal treatment temperature obtained from XRD.

Temperature	{101}	{103}	{004}	{112}
300 °C	12.1	11.0	15.0	14.1
400 °C	12.8	12.3	15.3	14.3
500 °C	14.5	15.0	16.4	16.7
600 °C	21.4	20.7	21.2	22.1
700 °C	28.8	29.5	27.6	26.8
800 °C	42.9	39.4	39.7	40.5
900 °C	49.0	37.3	45.2	44.8

Table S6: Crystallite size (nm) for the {101}, {103}, {004} and {112} planes for the sample TiO_2/IL 10% as a function of thermal treatment temperature obtained from XRD.

Temperature	{101}	{103}	{004}	{112}
As sint.	12.8	13.0	16.3	13.3
300 °C	12.5	11.0	15.5	18.2
400 °C	13.0	15.8	15.5	18.0
500 °C	14.4	15.4	16.2	17.8
600 °C	17.6	19.1	17.8	19.0
700 °C	25.2	24.6	25.0	27.5
800 °C	37.6	47.1	40.1	43.2
900 °C	47.5	40.1	41.4	40.2

Table S7: Anatase {112} plane % on the surface of the particles by Wulff construction.

Temperature	TiO_2	TiO_2/IL 1%	TiO_2/IL 10%
300 °C	19.37	11.4	0.0
400 °C	16.23	18.0	0.0
500 °C	30.81	16.9	8.352
600 °C	50.44	29.1	21.356
700 °C	49.05	51.5	22.222
800 °C	n.a.	43.0	22.876
900 °C	n.a.	43.7	50.252

Table S8: Surface contribution (%) to {101}, {103} and {004} planes for the sample TiO_2 as a function of thermal treatment temperature obtained from XRD.

Temperature	{101}	{103}	{004}
300 °C	60.5	18.5	6.5
400 °C	67.6	12.4	15.0
500 °C	58.5	0.00	42.6
600 °C	35.9	1.1	49.9
700 °C	33.1	3.8	56.1
800 °C	n.a.	n.a.	n.a.
900 °C	n.a.	n.a.	n.a.

Table S9: Surface contribution (%) to {101}, {103} and {004} planes for the sample TiO_2/IL 1% as a function of thermal treatment temperature obtained from XRD.

Temperature	{101}	{103}	{004}
300 °C	35.0	53.1	0
400 °C	37.8	43.8	1.1
500 °C	45.9	33.0	16.9
600 °C	32.1	32.9	23.5
700 °C	24.6	12.6	45.0
800 °C	13.3	37.1	26.1
900 °C	18.9	31.8	67.0

Table S10: Surface contribution (%) to {101}, {103} and {004} planes for the sample TiO_2/IL 10% as a function of thermal treatment temperature obtained from XRD.

Temperature	{101}	{103}	{004}
300 °C	51.9	48.1	0.00
400 °C	78.4	15.0	26.5
500 °C	56.7	34.0	3.5
600 °C	32.3	11.6	34.8
700 °C	36.8	35.2	23.4
800 °C	60.3	0.0	67.4
900 °C	11.3	32.4	24.1

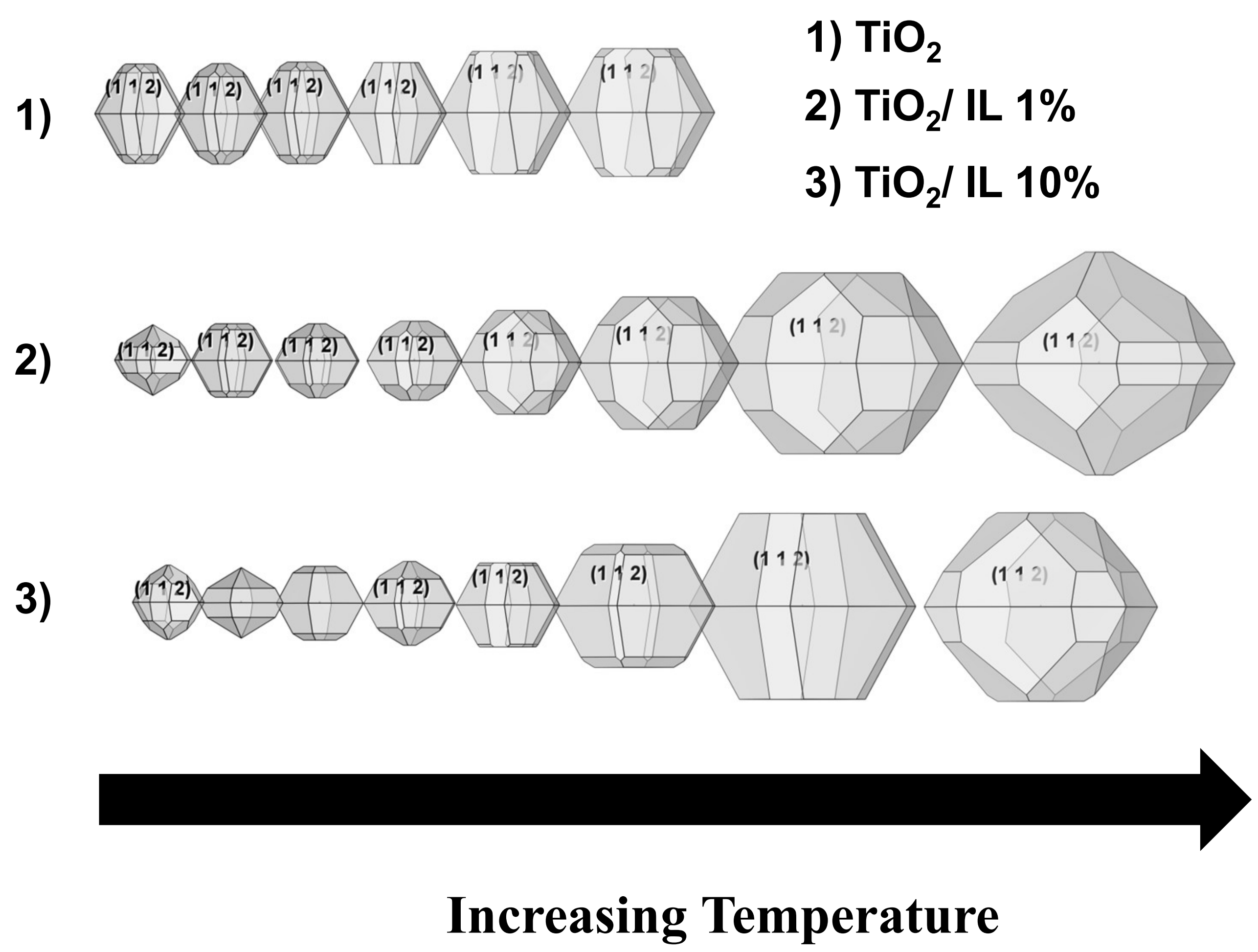

Figure S6: Scheme illustrating the morphology and size evolution of the anatase grains with increasing temperature obtained by Wulff construction.

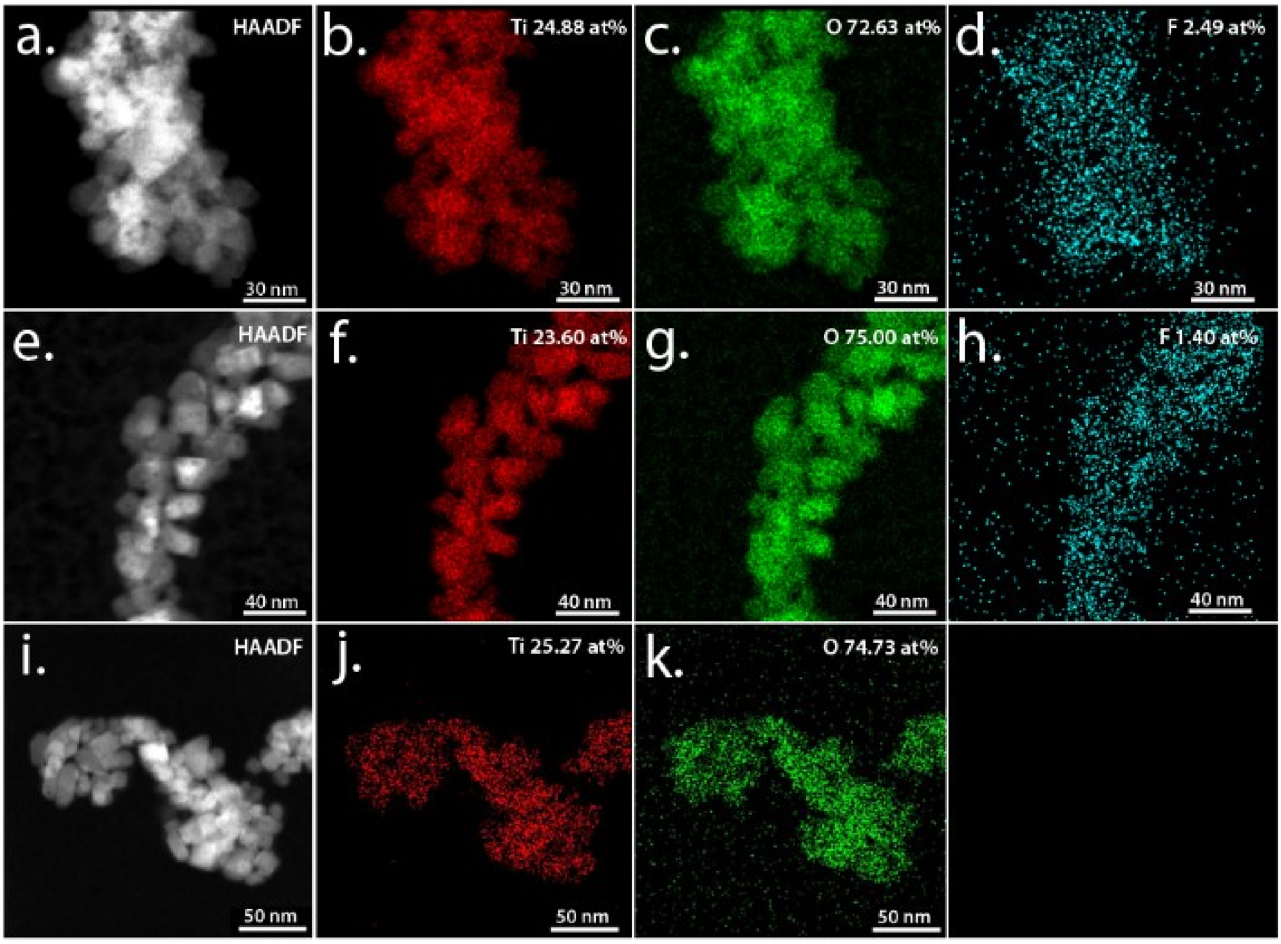

Figure S7: EDX mapping of TiO_2/IL 10% (a, b, c and d), TiO_2/IL 1% (e, f, g and h) and TiO_2 (i, j and k).

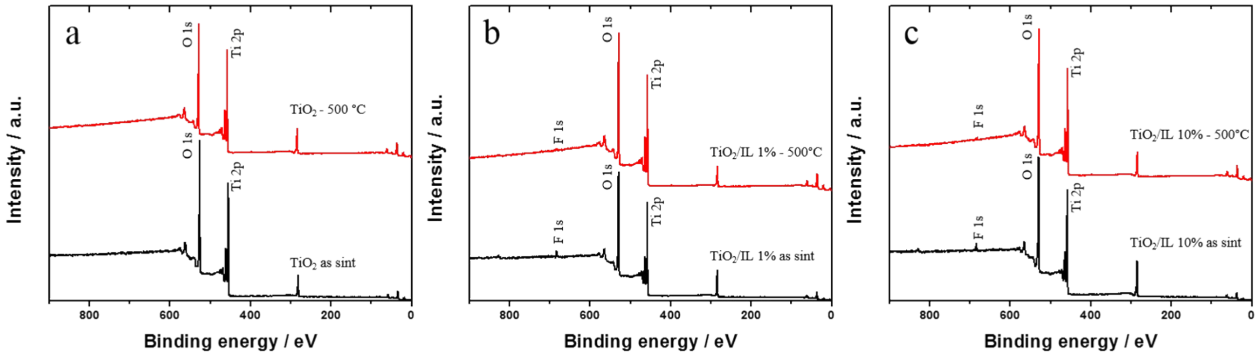

Figure S8: Survey spectra of the samples as synthetized and obtained after thermal treatment at 500 °C. (a) TiO_2, (b) TiO_2/IL 1% and (c) TiO_2/IL 10%.

Table S11: XPS analysis of Ti 2p, O 1s and F 1s for TiO_2, TiO_2/IL 1% and TiO_2/IL 10% obtained before and after thermal treatment at 500 °C.

samples	Atom / wt%		
	Ti 2p	O 1s	F 1s
TiO_2 – as synthetized	29.7	70.3	---
TiO_2 – 500 °C	30.9	69.1	---
TiO_2/IL 1% – as synthetized	30.2	67.2	2.4
TiO_2/IL 1% - 500 °C	30.2	68.7	1.1
TiO_2/IL 10% – as synthetized	29.2	67.9	2.9
TiO_2/IL 10% - 500 °C	28.4	70.0	1.6

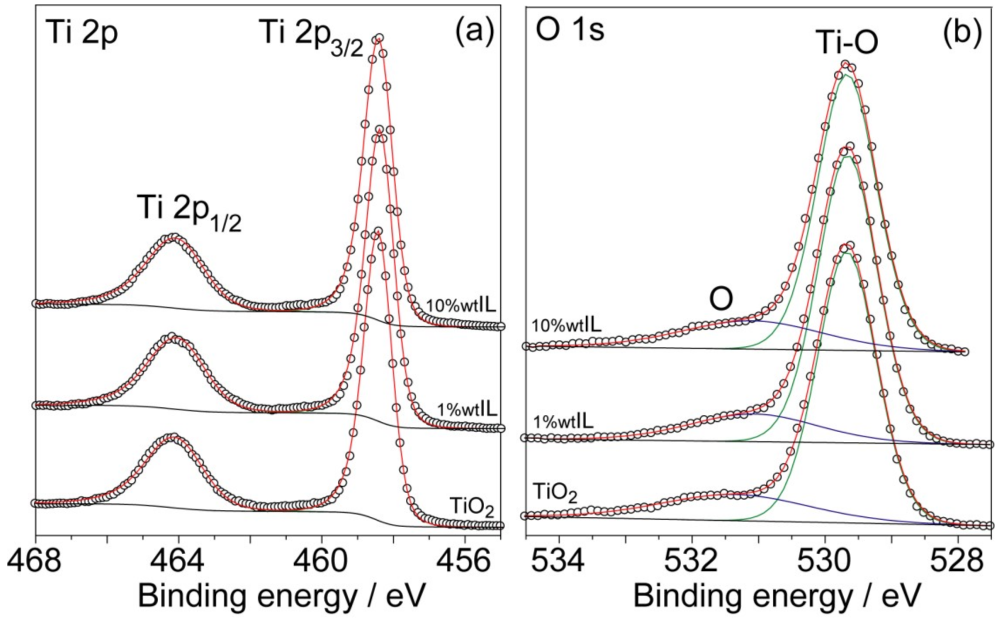

Figure S9: XPS spectra of (a) Ti 2p, (b) O 1s from the samples for TiO_2, TiO_2/IL 1% and TiO_2/IL 10% obtained before and after thermal treatment at 500 °C.

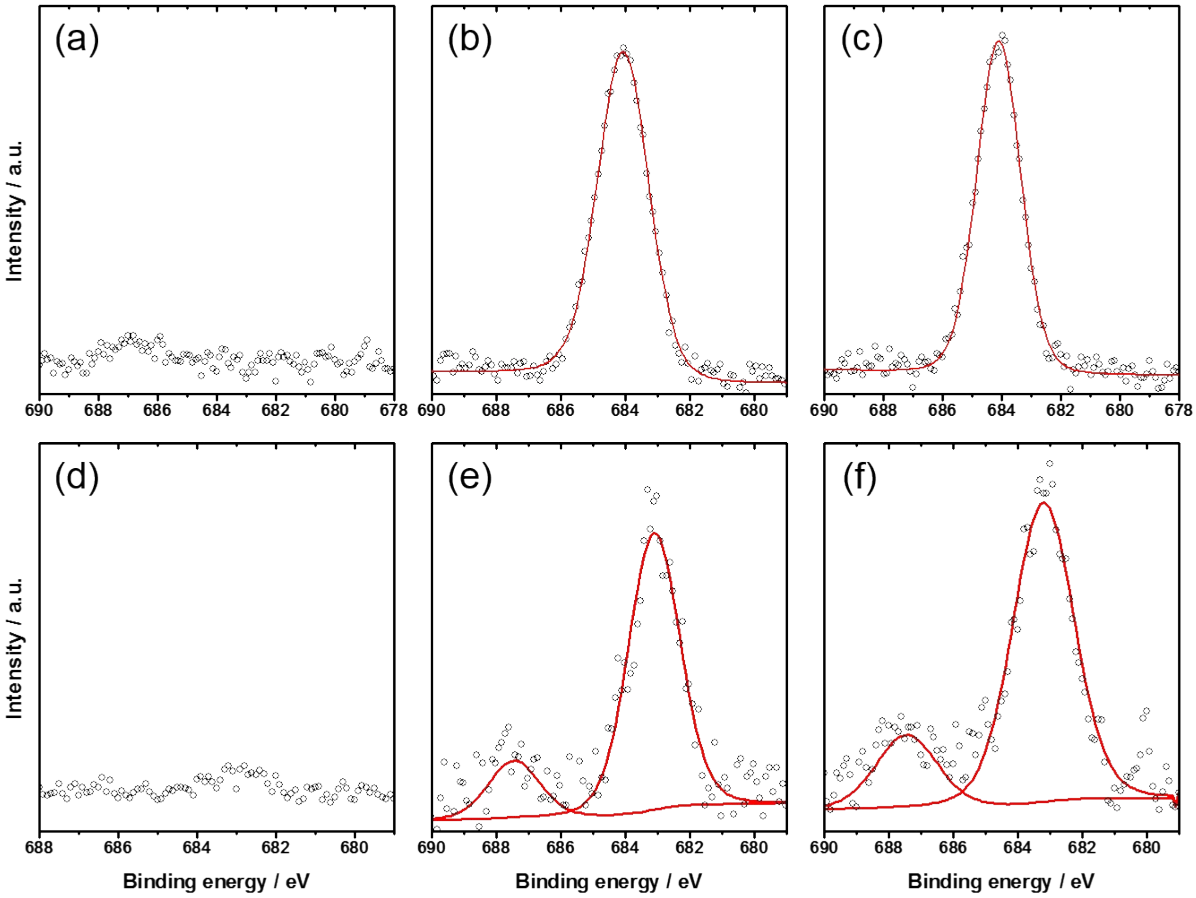

Figure S10: F 1s XPS spectra from as-synthesized TiO_2 (a) TiO_2/IL 1% (b) and TiO_2/IL 10% (c) and from the thermally treated samples at 500 ºC (d), (e) and (f) respectively.

Figure S11 shows the I-V curves of the solar cells assembled with mesoporous films of TiO_2, TiO_2/IL 1%, and TiO_2/IL 10% obtained after sintering at 500 ºC.

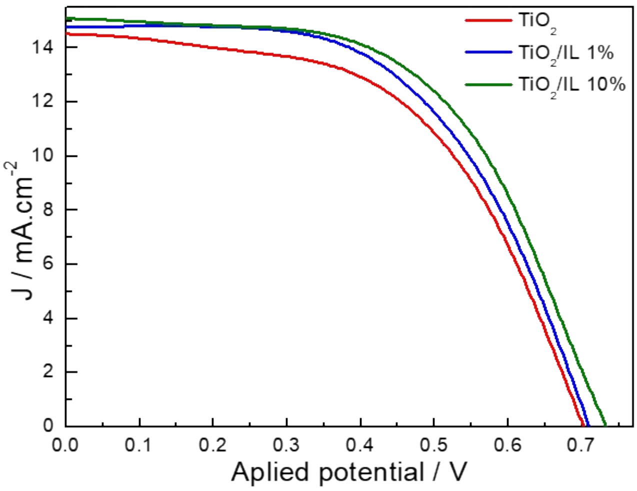

Figure S11. Photocurrent versus potential curves from DSSCs assembled with pristine TiO_2, TiO_2/IL 1%, and TiO_2/IL 10%.

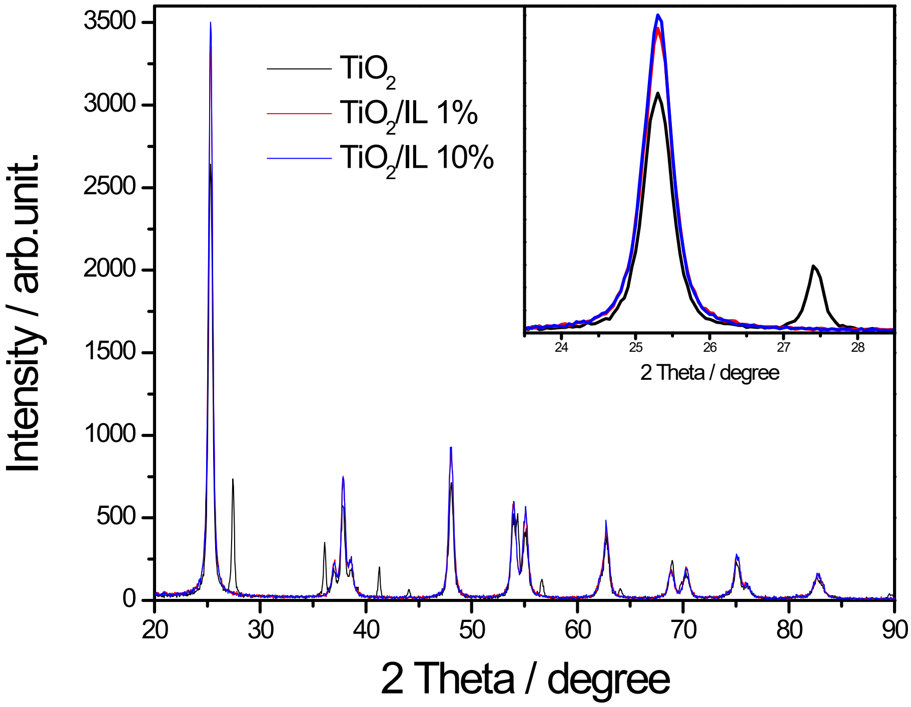

Figure S12. X-ray diffraction pattern for pristine TiO_2, TiO_2/IL 1%, and TiO_2/IL 10% after all sintering TiO_2 films fabrication for DSSCs devices assembled.

References

1 Momma, K.; Izumi, F. VESTA 3 for three-dimensional visualization of crystal, volumetric and morphology data. *J. Appl. Cryst.,* **2011**, 44, 1272–1276.

2 Rahimi, N.; Pax, R. A.; MacA. Gray, E. Review of functional titanium oxides. I: TiO_2 and its modifications. *Prog. Solid. State Ch.*, 2016, **44**, 86-105

3 Ito, S.; Murakami, T.; Comte, P.; Liska, P.; Gratzel, C.; Nazeeruddin, M.; Gratzel, M. Fabrication of thin film dye sensitized solar cells with solar to electric power conversion efficiency over 10%. *Thin Solid Films*, **2008**, 516, 4613-4619.

4 Parker, J. C.; Siegel, R. W. Raman microprobe study of nanophase TiO_2 and oxidation-induced spectral changes. *J. Mater. Res.*, **1990**, 5, 1246–1252.

5 Wang, J.; Rao, P.; An, W.; Xu, J.; Men, Y. Boosting photocatalytic activity of Pd decorated TiO_2 nanocrystal with exposed (001) facets for selective alcohol oxidations. *Appl. Catal. B*, **2016**, 195, 141–148.

6 Niu, M.; Cui, R.; Wu, H.; Cheng, D.; Cao, D. Correction to "Enhancement Mechanism of the Conversion Efficiency of Dye-Sensitized Solar Cells Based on Nitrogen-, Fluorine-, and Iodine-Doped TiO_2 Photoanodes". *J. Phys. Chem. C*, **2016**, 120, 3088.

7 Tang, J.; Quan, H.; Ye, J. Photocatalytic Properties and Photoinduced Hydrophilicity of Surface-Fluorinated TiO_2. *Chem. Mater.*, **2007**, 19, 116−122.
